# Improvement of morning headache in adults with obstructive sleep apnea after positive airway pressure therapy

**DOI:** 10.1038/s41598-023-34896-0

**Published:** 2023-09-05

**Authors:** Min Young Seo, Min Kyu Lee, Mun Soo Han, Jun Yoo, Seung Hoon Lee

**Affiliations:** Division of Rhinology and Sleep Medicine, Department of Otorhinolaryngology-Head and Neck Surgery, Korea University Ansan Hospital, Ansan, Korea University College of Medicine, 123, Jeokgeum-ro, Danwon-gu, Ansan-si, Gyeonggi-do 15355 South Korea

**Keywords:** Respiratory tract diseases, Respiratory signs and symptoms

## Abstract

The aim of this study was to evaluate the association between obstructive sleep apnea and morning headache and to assess the improvement of morning headache following positive airway pressure therapy. One hundred and sixteen participants were enrolled in this study; all of them received positive airway pressure therapy for at least 3 months. We checked the differences in various sleep apnea-related parameters according to the presence of morning headache and evaluated the improvement of morning headache following positive airway pressure therapy. Among the 116 study participants, 103 were men, with a mean age and body mass index of 50.34 ± 10.23 years and 28.00 ± 4.21 kg/m^2^, respectively. The severity of morning headache was higher in the severe obstructive sleep apnea group than in the mild to moderate group (2.16 ± 1.70 vs. 1.50 ± 1.57, *P* = 0.027). However, the various polysomnographic parameters did not significantly differ according to the presence of headache. The Epworth sleepiness scale score was significantly higher in the morning headache presence group than in the absence group (10.90 ± 5.45 vs. 8.13 ± 4.27, *P* = 0.003). Furthermore, a notable correlation was observed between the reduction in daytime sleepiness and the improvement in morning headache following PAP treatment (r = 0.503, *P* < 0.001). Morning headache significantly improved following positive airway pressure therapy (prevalence: 53.4–16.4%; severity: 1.92 ± 1.67 vs. 0.86 ± 0.80, all *P* < 0.001), especially in the patients with morning headache before positive airway pressure therapy. Morning headache is significantly associated with daytime sleepiness and positive airway pressure therapy improves morning headache.

## Introduction

Obstructive sleep apnea (OSA) is a common disorder, with a prevalence of 22% (9–37%) in men and 17% (4–50%) in women^1^. It is characterized by repetitive upper airway obstruction during sleep, hypoxia, and hypercapnia. These events activate the sympathetic nervous system and eventually lead to various diseases, including cardiovascular, metabolic, neurologic, and psychological problems^2–6^. Thus, appropriate treatment is essential for the prevention of various morbidities associated with OSA. Positive airway pressure (PAP) therapy is the standard first-line treatment for OSA, especially in patients with moderate to severe OSA^7,8^; it improves patients’ quality of life, excessive daytime sleepiness (EDS), and various morbidities^9–11^.

In our experience, many patients with OSA complain of morning headache in outpatient clinics, and several clinical studies have reported the prevalence of morning headache in patients with OSA from 15.2 to 74%^12,13^. When we consider the prevalence of morning headache in the general population (5–7%)^13^, it can be suggested that OSA and morning headache are significantly associated. Furthermore, a number of patients with OSA accompanied by morning headache reported improvements of headache after PAP therapy. However, the causal relationship between various sleep apnea-related parameters and morning headache is controversial^14–17^. In addition, the relief of morning headache after PAP therapy is also debatable^12,18^.

The aim of this study was to evaluate the association between various sleep apnea-related parameters and morning headache and to assess morning headache improvement before and 3 months after PAP therapy.

## Results

Among the 116 study participants, 103 were men, with a mean age and body mass index of 50.34 ± 10.23 years and 28.00 ± 4.21 kg/m^2^, respectively. The prevalence of morning headache in our study subjects was 53.4% (62/116) before PAP therapy. In the comparison of the severity and prevalence of morning headache according to OSA severity, we found that the severity of morning headache was significantly higher in the severe OSA group than in the mild to moderate OSA group. Furthermore, the prevalence of morning headache was also higher in the severe OSA group than in the mild to moderate OSA group; however, this difference was not significant (Table 1).

**Table 1:** Demographic data of study subjects and severity and prevalence of morning headache according to OSA severity.

	Mild to moderate OSA (n = 42)	Severe OSA (n = 74)	*P* value
Age	52.24 ± 9.16 (53, 45–58)	49.27 ± 10.70 (51, 42–56)	0.134^†^
Sex (M:F)	37:5	66:8	> 0.999^§^
BMI (kg/m^2^)	26.23 ± 2.83 (26, 24.8–27.4)	29.00 ± 4.53 (28.2, 25.8–31.7)	**< 0.001**^†^
AHI (/h)	18.41 ± 6.59 (18.7, 14.4–22.3)	58.45 ± 19.39 (57.6, 42.8–69.0)	**< 0.001**^†^
Arousal index (/h)	35.59 ± 15.29 (34.1, 25.2–43.4)	61.59 ± 17.98 (61.4, 50.6–69.3)	**< 0.001**^‡^
Minimal SaO_2_ (%)	77.08 ± 11.15 (78.5, 74–84)	66.61 ± 10.92 (68, 57–76)	**< 0.001**^‡^
Morning headache severity (Likert scale, 0–6)	1.50 ± 1.57 (1, 0–2)	2.16 ± 1.70 (2, 1–3)	**0.027**^‡^
Prevalence of morning headache (%)	42.9 (18/42)	59.5 (44/74)	0.121^§^

Independent T-test^†^, Mann–Whitney U test^‡^, Chi-square test^§^

OSA; obstructive sleep apnea, BMI; body mass index, AHI; apnea–hypopnea index.

Data are mean ± standard deviation (median, Q1–Q3).

Statistical significance level of* P* < 0.05, Bold values indicate statistically significant.

**Figure 1:**
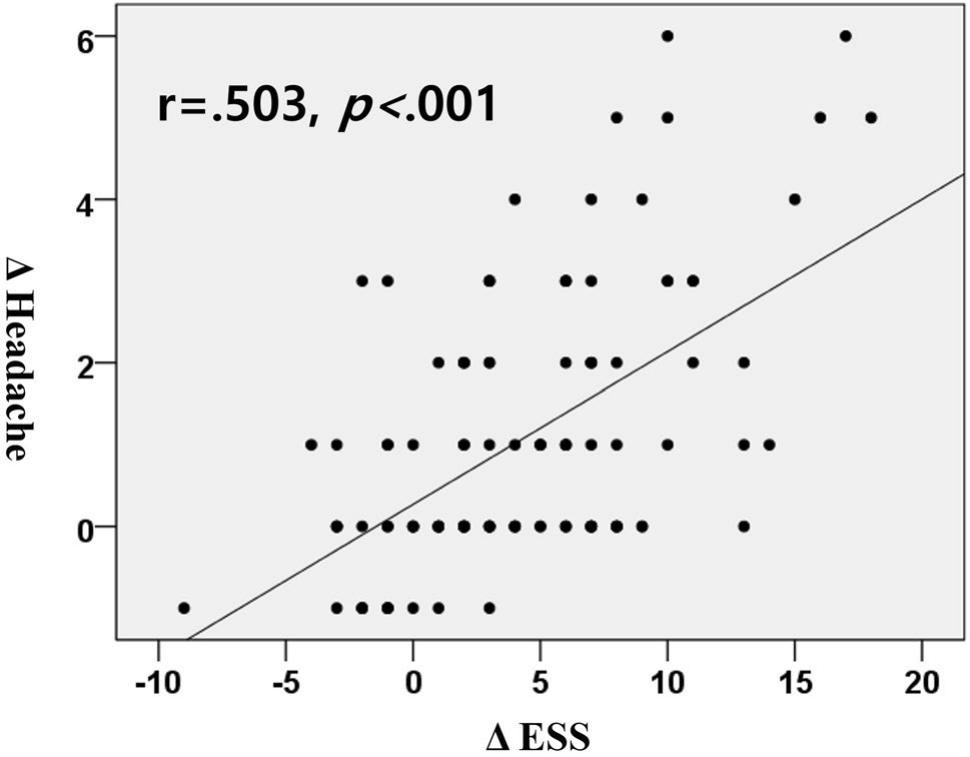
Correlation coefficiency between degree of improvement of morning headache and ESS score. Spearman’s correlation method was performed to evaluate the relationship between both parameters.

We compared the various parameters of polysomnography (PSG) and demographics according to the presence of morning headache and did not find any differences between the two groups. However, the ESS score was significantly higher in the morning headache group (Table 2). In the evaluation of changes in morning headache after PAP therapy, we found that the prevalence of morning headache significantly decreased after PAP therapy from 53.4 to 16.4%. Moreover, the severity of morning headache significantly decreased with PAP therapy. When we evaluated in the group with morning headache before PAP therapy, the severity of morning headache significantly decreased following PAP therapy (3.15 ± 1.34 to 1.13 ± 0.86, *P* < 0.001), and improvement of morning headache was observed in both the mild to moderate and severe OSA groups. Particularly, among the patients with severe sleep apnea with morning headache initially, the severity of morning headache improved by 72% after PAP therapy (3.20 ± 1.41 to 0.95 ± 0.71, *P* < 0.001). Therefore, we suggest that the improvement of morning headache was significantly greater when only those with headache were analyzed (Table 3). Furthermore, although there was no statistical significance in the improvement of headaches according to PAP compliance, it was observed that the good compliance group (n = 92) showed a greater improvement in headaches (0.83 ± 1.55 vs 1.12 ± 1.66, *P* = 0.447) compared to the poor compliance group (n = 24). Additionally, when analyzing whether there was a significant correlation between the improvement in daytime sleepiness measured by Epworth sleepiness scale (ESS) and the improvement in morning headache after PAP treatment, it was found that there was a statistically significant correlation (Fig. 1).

**Table 2:** Various parameters as morning headache presence.

	Morning headache (−) (n = 54)	Morning headache (+) (n = 62)	*P* value
Sex (M:F)	51:3	52:10	0.084^§^
BMI (kg/m^2^)	28.13 ± 4.36 (27.3, 25.6–30.4)	27.89 ± 4.10 (27.1, 24.5–30.5)	0.757^†^
AHI (/h)	42.56 ± 27.90 (38.4, 19.9–61)	45.16 ± 22.45 (42.4, 25.5–63.7)	0.321^‡^
RDI (/h)	50.20 ± 24.56 (51.1, 31.1–63.7)	53.07 ± 20.14 (53.6, 39.6–67.9)	0.492^†^
Arousal index (/h)	51.54 ± 23.65 (51.3, 33.1–63.4)	52.72 ± 18.82 (52.7, 37.9–66.2)	0.766^†^
Minimal SaO_2_ (%)	75.62 ± 13.17 (76, 66–81)	69.34 ± 11.01 (70, 60–78)	0.128^‡^
ODI (/h)	40.43 ± 28.82 (39.9, 16.3–61.8)	42.01 ± 23.18 (41.7, 19.7–62.2)	0.744^†^
Sleep efficiency (%)	86.51 ± 10.05 (89, 83.1–93.9)	86.92 ± 8.39 (88.2, 82.4–93.4)	0.812^‡^
Total sleep time (min)	397.61 ± 51.59 (396, 360–425)	386.47 ± 43.36 (400, 364–425)	0.952^‡^
Stage R sleep (%)	15.98 ± 6.32 (15.1, 11.1–19.7)	15.25 ± 5.58 (15.6, 9.8–21.8)	0.861^‡^
ESS	8.13 ± 4.27 (7, 5–11)	10.90 ± 5.45 (11, 7–14)	**0.003**^†^

Independent T-test^†^, Mann–Whitney U test^‡^**,** Chi-square test^§^

*BMI* body mass index, *RDI* respiratory disturbance index, *AHI* apnea–hypopnea index, *ODI* oxygen desaturation index, *ESS* Epworth sleepiness scale.

Data are mean ± standard deviation (median, Q1–Q3).

Statistical significance level of* P* < 0.05, Bold values indicate statistically significant.

**Table 3:** Improvement of morning headache in OSA patients with morning headache as PAP therapy.

	Pre PAP (Likert scale, 0–6)	Post PAP 3 months (Likert scale, 0–6)	*P* value
Mild to moderate OSA (n = 18)	3.00 ± 1.19 (2.5, 2–4)	1.56 ± 1.04 (1, 1–2)	**< 0.001**
Severe OSA (n = 44)	3.20 ± 1.41 (3, 2–4)	0.95 ± 0.71 (1, 0–1)	**< 0.001**
Total (n = 62)	3.15 ± 1.34 (3, 2–4)	1.13 ± 0.86 (1, 1–2)	**< 0.001**

Mann–Whitney U test.

*PAP* positive airway pressure, *OSA* obstructive sleep apnea.

Data are mean ± standard deviation (median, Q1–Q3).

Statistical significance level of* P* < 0.05, Bold values indicate statistically significant.

## Discussion

In our study participants with OSA, the prevalence of morning headache was reported as 53.4%, and various demographics and polysomnographic parameters did not significantly differ according to the presence of morning headache. To date, several studies have reported an association between morning headache and sleep apnea-related parameters. In a previous large population-based study, Kristiansen et al. reported that the average and lowest oxygen saturation levels during sleep were not significantly different according to the presence of morning headache, and Sand et al. also reported that there was no relationship between headache and oxygen desaturation^17,19^. Moreover, Lovati et al. reported that better respiratory parameters (higher mean oxygen saturation and lower time with an oxygen saturation lower than 90%) were found among headache sufferers with respect to those without headache^20^. When we consider the results of these studies, hypoxia and respiratory events were not sufficient factors for explaining the mechanism of morning headache. Therefore, we should perform further evaluation to reveal the pathophysiologic mechanism, including the factors related to elevated intracranial pressure^21^. We consider that basic research is needed to suggest the candidate factors associated with causal relationships.

In this study, we found that daytime sleepiness was significantly associated with morning headache. The ESS is a questionnaire used for the assessment of daytime sleepiness (0–24 points), and the sum of each scale number from 10 to 24 reflects EDS^22–24^. In our analysis, the mean ESS score was 10.90 ± 5.45 in the morning headache presence group and 8.13 ± 4.27 in the morning headache absence group. Recently, Kristoffersen et al. reported that increased EDS was associated with a higher headache frequency^25^. In addition, Kim et al. also reported that the prevalence of EDS was higher among subjects with chronic tension-type headache (TTH), and those with TTH with EDS had a higher frequency and intensity of headache than those without EDS^26^. They also reported that EDS is a disabling condition caused by poor nocturnal sleep, which can lead to daytime dysfunction, and is significantly associated with TTH exacerbation^26^. Thus, we considered that patients with morning headache also experience EDS, and treatment for EDS may be helpful in improving morning headache. According to the recommendations of the Standards of Practice Committee and the Board of Directors of the AASM, continuous PAP is indicated for improving self-reported sleepiness in patients with OSA, and the recommendation grade was suggested as the standard^27^. According to the definition of the recommendations, the term “standard” reflects a high degree of clinical certainty and generally implies the use of level I evidence^27^. Therefore, we considered that PAP therapy might also improve morning headache and then assessed changes in morning headache after PAP therapy.

To our knowledge, this study is the largest study (n = 116) that compared morning headache after PAP therapy among patients with OSA. Herein, we found that the prevalence and severity of morning headache significantly improved with PAP therapy. Several studies have reported the improvement of headache after PAP therapy in patients with OSA. Goksan et al. reported that among 76 patients treated with PAP therapy, morning headache was totally resolved in 70 patients after 1 month of treatment^12^. Furthermore, Johnson et al. also performed a retrospective study in 52 patients with OSA and concluded that treating OSA with PAP therapy improves headache in some patients^18^. We also found another study that concluded that PAP therapy alone did not improve headache. However, the authors also mentioned that their study sample size was fairly small (n = 21); thus, further investigation is needed^28^.

This study has several limitations. First, owing to the retrospective nature of this study, we could only find an association between EDS and morning headache. We could not determine the causal relationship between EDS and morning headache and could not suggest the pathophysiologic mechanism underlying morning headache. Thus, additional basic studies including cytokine or genetic analyses of study subjects are needed to provide more valuable data on this topic. Second, we evaluated morning headache among subjects with OSA but found that morning headache was not always associated with OSA. Furthermore, according to the International Classification of Headache Disorders third edition, sleep apnea-related headache was classified separately from morning headache^29^. Because of the retrospective design of this study, we do not have sufficient data on the characteristics of headache for the diagnosis of sleep apnea-related headache. We consider that if the assessment was performed using the sleep apnea-related headache criteria, we might have found associations with various PSG parameters. Therefore, we will conduct an additional prospective study on various PSG parameters and sleep apnea-related headache. Third, due to the majority of participants being male in this study, statistical analysis based on gender differences could not be performed. However, previous studies have reported that the incidence of morning headache is about twice as high in women compared to men^19^. Therefore, it is important to always consider gender differences when interpreting the results of this study.

## Conclusions

Morning headache is a common symptom in patients with OSA. Herein, the severity was higher in the severe OSA group than in the mild to moderate OSA group. Although we could not find an association between morning headache and various PSG parameters, subjective daytime sleepiness was significantly associated with morning headache. We found that short-term PAP therapy can improve morning headache in adult patients with OSA. Moreover, it can improve the severity of morning headache by 72% in patients with severe OSA. Therefore, when patients with OSA complain of morning headache, PAP therapy may be used as a fundamental treatment modality, which can reduce the need for medication.

## Methods

### Participants

We retrospectively reviewed the medical records of 116 patients with OSA who received PAP therapy consecutively for at least 3 months from December 2017 to June 2020. All of the study subjects underwent endoscopic and computed tomographic examination of the upper airway and full-night PSG for the evaluation and diagnosis of OSA. In addition, all participants completed symptom questionnaires, including the ESS, for the assessment of daytime sleepiness at the initial visit and before and 3 months after PAP therapy. The patients with neurological disorders or those regularly taking analgesics were excluded during the initial data collection phase. In our institutes’ clinical setting, when the patients were diagnosed with OSA based on the PSG findings and the physician decided the treatment modality as PAP therapy, PAP titration was conducted manually by well-trained sleep technicians at the sleep study center of our tertiary hospital and reviewed by certified sleep physicians. The physician prescribed the auto PAP device (DreamStation AutoCPAP, Philips Respironics, Murrysville, PA, USA) according to the optimal pressure range (optimal pressure ± 2 cmH_2_O). The patients visited our outpatient clinic at 2 weeks, 5 weeks, and 3 months after PAP initiation, and the PAP device was repeatedly set up for appropriate treatment. The compliance to PAP therapy was also assessed at 3 months after PAP therapy initiation; good compliance was defined as using a PAP device for ≥ 4 h daily and ≥ 70% of nights^30^. This study was approved by the Institutional Ethics Committee of Korea University Ansan Hospital and informed consent is waived due to the nature of the study, according to the approval of the Institutional Review Board (2020AS0258). All research was performed in accordance with the Declaration of Helsinki.

### Polysomnographic evaluation

Full-night PSG was performed using an Alice 6 device (Respironics, Murrysville, PA, USA) in our tertiary hospital using the standard American Academy of Sleep Medicine (AASM)-recommended neurophysiologic and respiratory signals, electroencephalography, electromyography, electrooculography, and electrocardiography. Oro-nasal airflow was detected using a thermistor for apnea detection and a pressure transducer for hypopnea detection. Chest and abdominal wall movements were also measured using plethysmography, and oxygen saturation was measured using pulse oximetry. Polysomnographic data were manually scored by a well-trained sleep technician and reviewed by certified clinical physicians according to the recommended AASM criteria^31^.

Apnea and hypopnea were defined as > 90% reduction of airflow and ≥ 30% reduction of airflow with a decrease in SpO_2_ of ≥ 4% or arousal for at least 10 s, respectively. We described OSA as an apnea–hypopnea index (AHI) of ≥ 5 and classified it by severity into mild to moderate (AHI: 5–30) and severe (AHI: ≥ 30) OSA groups.

### Assessment of headache

All participants completed a questionnaire on the severity of morning headache over the past four weeks using a 7-point Likert scale (0–6 points) before and 3 months after PAP initiation. For the assessment of the prevalence of morning headache as a categorical variable, the subjects who checked points 0 and 1 were considered to have no morning headache.

### Statistical analysis

Statistical analysis was performed using the Statistical Package for the Social Sciences version 21 (IBM Corporation, Armonk, NY, USA). An independent t-test or the Mann–Whitney U test was performed on the basis of the data distribution to compare the demographics of the patients and to evaluate the association between the presence of headache and various PSG parameters. The chi-square test or Fisher’s exact test was performed to compare the prevalence of morning headache according to OSA severity and PAP therapy. Spearman’s correlation coefficient was used to assess the relationship between improvement of headache and improvement of daytime sleepiness according to ESS score.

### Ethics approval

This study was approved by the Institutional Ethics Committee of Korea University Ansan Hospital (2020AS0258).

## Data Availability

The datasets generated and analyzed during the current study are available from the corresponding author on reasonable request.
